# Lung function predicts mortality in people with serious mental illness: A 6-year follow-up study

**DOI:** 10.1192/j.eurpsy.2025.10138

**Published:** 2025-12-03

**Authors:** Maria Jose Jaen-Moreno, David Laguna-Muñoz, Gloria Isabel del Pozo, Cristina Camacho-Rodríguez, Ipek Guler, José Angel Alcalá, Micaela Reyes-López, Montserrat Alcántara, Diego Benítez-Jurado, Cristina Ruiz-Rull, Ana Jiménez-Peinado, Nuria Feu, Eduard Vieta, Vicent Balanzá-Martínez, Fernando Sarramea

**Affiliations:** 1 Maimonides Biomedical Research Institute of Cordoba (IMIBIC), Cordoba, Spain; 2 University of Cordoba, Department of Morphological and Sociosanitary Science, Córdoba, Spain; 3 Reina Sofia University Hospital, Córdoba, Spain; 4 Primary care center Cruz de Caravaca, Almería, Spain; 5 Department of Medicine, School of Medicine & Health Sciences, University of Barcelona (UB), 143 Casanova st., 08036 Barcelona, Catalonia, Spain; 6 Bipolar and Depressive Disorders Unit, Hospìtal Clinic, 170 Villarroel st., 08036 Barcelona, Catalonia, Spain; 7 Institut d’Investigacions Biomèdiques August Pii Sunyer (IDIBAPS), 170 Villarroel st., 08036 Barcelona, Catalonia, Spain; 8 Institute of Neurosciences (UBNeuro); 9 Centro de Investigación Biomèdica en Red de Salud Mental (CIBERSAM), Instituto de Salud Carlos III, Madrid, Spain; 10 Unitat Docent de Psiquiatría i Psicología Médica, Departament de Medicina, Universitat de València, CIBERSAM, Valencia, Spain

**Keywords:** bipolar disorder, lung function, mortality, schizophrenia, serious mental illness

## Abstract

**Background:**

The population with a serious mental illness (SMI) shows a high risk of premature mortality. Overexposed to multiple health risks throughout life, their main threat is physical illness, which starts earlier and is not diagnosed in time. Developing preventive actions is a public health priority.

**Methods:**

This longitudinal prospective study evaluated the predictive value of lung function on all-cause mortality in patients with schizophrenia (SCHIZ) or bipolar disorder. Patients aged 40–70 years, active smokers, and without preexisting respiratory disease underwent spirometry following American Thoracic Society/European Respiratory Society 2021 standards. Mortality data were collected through December 2022. Cox proportional hazards models and Kaplan–Meier survival curves analyzed the association between lung function, specifically forced expiratory volume in the first second (FEV1), forced vital capacity (FVC), and mortality, adjusting for relevant confounders (age, gender, abdominal circumference, and comorbidities).

**Results:**

Of 107 participants (mean age 49.3 years, 63.3% male) with SMI (72% SCHIZ) and active smokers, 8 (7.5%) died during the 6-year follow-up (5 cardiovascular and 3 cancer). Mean *z*-scores were −1.41 (SD = 1.22) for FEV1 and −0.99 (SD = 1.16) for FVC. Lower FEV1 and FVC *z*-scores were significantly associated with increased mortality risk (*p* = 0.002 and *p* = 0.009, respectively). Kaplan–Meier analysis confirmed this association for FEV1 (*p* = 0.039) and FVC (*p* = 0.007) but not for gender, comorbidities (hypertension, diabetes, and dyslipidemia), or FEV1/FVC. A multivariate Cox regression model, adjusting for age and abdominal circumference, confirmed the independent predictive value of lower FEV1 *z*-score for mortality (hazard regression = 0.473, 95% confidence interval: 0.220–0.979, *p* = 0.044).

**Conclusions:**

Poorer lung function, especially lower FEV1, was independently associated with all-cause mortality in SMI. Spirometry, an easily implementable technique, could help to detect at-risk individuals and favor prevention initiatives.

## Introduction

Living with a serious mental illness (SMI), such as bipolar disorder (BD) or schizophrenia (SCHIZ), is associated with a reduction in life expectancy of 12–15 years [1]. Despite the high risk of suicide, in SMI, most deaths and years of life lost are due to natural causes [2]. Cardiovascular, metabolic, and respiratory diseases, present at a younger age and with a generally worse prognosis, are the main threat [3, 4]. These patients are exposed to multiple risk factors at different levels: individual factors, healthcare systems, and social determinants of health [5].

Most of the meta-research on physical health in this population has focused in recent years on cardiovascular and metabolic risks. However, data from different European and US cohorts highlight a high risk of respiratory mortality, especially in young populations [6, 7]. Among individuals with SMI, the smoking rate – a key critical factor for respiratory disease – is up to three times higher than that of the general population, with greater levels of dependence and a lower probability of quitting [8, 9]. Recent meta-research, based on clinical samples and population databases, shows an association between SMI and the most frequent respiratory diseases [10, 11], as well as a higher likelihood of altered respiratory parameters in spirometry [11–13].

Lung function is considered a marker of overall physical health [14]. Spirometry is the gold standard for studying lung volumes in a simple, inexpensive, and noninvasive way [15]. The test defines several expiratory flow volumes: forced expiratory volume in the first second (FEV1), forced vital capacity (FVC), and the ratio between them (FEV1/FVC), all of which are associated with the risk of premature mortality in general population samples [16]. Recently, the usefulness of this test for detecting individuals at risk and developing prevention measures has been highlighted [17].

As part of the longitudinal follow-up of a clinical trial designed for smoking cessation in people with SMI, we prospectively studied a sample of smokers with BD and SCHIZ. For the first time in individuals with SMI, and based on the hypothesis that respiratory function can help identify those at special risk in populations with a high probability of premature mortality, we evaluated the ability of different baseline lung volumes, measured by spirometry, to predict mortality in people with SMI.

## Patient and methods

### Design

This longitudinal, prospective, observational study was conducted at eight community mental health centers in Andalucia (southern Spain). The Reina Sofía Hospital Ethics Committee, Córdoba, approved the study (reference 4883, act no. 320).

### Study population

In 2017, patients were recruited to participate in a randomized controlled trial (RCT) to evaluate the effect of a motivational tool for smoking cessation (see Jaen-Moreno et al. [18]).

Patients were included in the trial if they met the following inclusion criteria: (1) age between 40 and 70 years; (2) diagnosed with SCHIZ or BD (as per Diagnostic and Statistical Manual of Mental Disorders, 4th Edition, Text Revision criteria); (3) active smokers (current consumption of at least 10 cigarettes per day with more than 10 packs/year for a cumulative consumption); and (4) clinical stability, defined as a Hamilton Rating Scale for Depression [19] score < 14, a Young Mania Rating Scale [20] score < 6, or a Positive and Negative Syndrome Scale [21] score < 70 points. We excluded patients if they had (1) a current or previous respiratory disease, (2) a diagnosis of any pathology that contraindicates the performance of spirometry, and (3) a current psychiatric or cognitive state that significantly impaired ability to understand and follow spirometry instructions.

### Study procedures

We offered follow-up under clinical practice conditions for patients who participated in the RCT and had validated spirometry results. We collected baseline data on sociodemographics, cardio-metabolic comorbidities (hypertension, diabetes, and dyslipidemia), key predictors of mortality [22], psychiatric conditions, anthropometrics (abdominal circumference), vital signs, smoking habits, and physical activity. We assessed physical activity using the International Physical Activity Questionnaire [23], which allows us to calculate an activity index (based on metabolic equivalents of task [METS]) and a sedentary index. We conducted spirometry evaluations at each participant’s site using the DATOSPIR Touch Easy D+ spirometer (Sibelmed, Barcelona, Spain). The head of the Functional Test Unit in the Pneumology Service at Reina Sofia Hospital (Córdoba, Spain) assessed all spirometry measurements.

We conducted a mortality analysis with a data cutoff on December 31, 2022, using medical records to identify patients who died and the causes of death during the follow-up period.

### Spirometry procedure

The spirometry procedures followed the American Thoracic Society/European Respiratory Society (ATS/ERS) standardization criteria [24] for equipment validation, quality control, acceptability, and repeatability. A maximum of eight maneuvers was performed until three acceptable maneuvers were achieved. Reversibility testing involved repeating three maneuvers 15 min post-bronchodilation with salbutamol. FVC, FEV1, and FEV1/FVC were measured. Personnel were trained by the pulmonology service of Reina Sofía Hospital (Córdoba, Spain) and underwent accredited training covering theoretical principles, equipment handling, and spirometry performance [25]. Following the 2021 ATS and ERS standards [26], *z*-scores were calculated using the Global Lung Initiative reference equations, which are based on standardized values derived from the general population. Based on this procedure, the lower limit of normal (LLN) represents the cutoff values that fall outside the normal range (5th percentile or −1.645). The severity of lung function – FEV1, FVC, and the ratio FEV1/FVC (for all measures using the *z*-score) – was considered mild (−1.65 to −2.5), moderate (−2.51 to −4.0), or severe (<−4.1).

### Statistical analyses

Descriptive statistics were calculated for all variables. Normally distributed continuous variables were reported as means and standard deviations (SDs), while non-normally distributed variables were presented as medians and interquartile ranges. Categorical variables were expressed as frequencies and percentages.

Kaplan–Meier survival curves, with mortality as a dependent variable, were used to represent the differences between groups based on gender, the presence of cardio-metabolic comorbidities (hypertension, diabetes, and dyslipidemia), and severity categories (mild, moderate, and severe) for the lung function parameters. These differences have been statistically tested by using the log-rank test.

Furthermore, univariate Cox proportional hazards regression (HR) analyses were performed to estimate potential risk factors and mortality associations. Variables included in the univariate analyses were age, gender, smoking status (pack-years), abdominal circumference (cm), presence of comorbidities (hypertension, diabetes, and dyslipidemia), FEV1 *z*-score, FVC *z*-score, and FEV1/FVC ratio *z*-score.

Two separate multivariate Cox regression models were constructed to address potential multicollinearity and adjust for confounders. Variables that were significant (*p* < 0.05) in the univariate analysis or exhibited trends (*p* < 0.1) were included in the regressions.

All statistical analyses were performed using SPSS version 28. A two-sided *p*-value of < 0.05 was considered statistically significant.

## Results

Our study included 107 participants diagnosed with SMI in the analysis. The mean age of the sample was 49.3 years, and 63.3% were male. Seventy-two percent were diagnosed with SCHIZ, and all participants were active smokers. During the follow-up, eight participants had a natural death. Three cases were attributed to oncological causes, while five were to cardiovascular ones. About the comorbidities, 13.1% of the sample had been diagnosed with diabetes, 17.8% had dyslipidemia, and 10.3% had hypertension. Regarding lung function parameters, *z*-scores for FEV1, FVC, and FEV1/FVC were −1.41 (1.22), −0.99 (1.16), and 0.88 (0.99), respectively (Table 1).

**Table 1.** Demographic and clinical characteristics

	Overall (*n* = 107)	Deceased (*n* = 8)	Survivor (*n* = 99)	*p*
Age, mean (SD)	49.29 (5.95)	52.75 (4.92)	40.02 (5.96)	0.063
Gender, *n* (%)				0.708
Female	39 (36.4)	2 (25)	37 (37.4)	
Male	68 (63.3)	6 (75)	62 (62.6)	
Diagnosis, *n* (%)				1.000
Schizophrenia	77 (72)	6 (75)	71 (71.7)	
Bipolar disorder	30 (28)	2 (25)	28 (28.3)	
Pack/year, mean (SD)	38.48 (18.16)	42.03 (19.63)	36.03 (18.07)	0.359
Cigarettes per day, mean (SD)	23.41 (10.53)	22.75 (9.93)	23.46 (10.62)	0.918
Abdominal circumference, mean (SD)	106.81 (15.24)	120.88 (23.67)	105.61 (13.92)	0.051
Physical activity (IPAQ), median (IQR)				
Activity (METS)	693 (198–1386)	247 (0–1806.75)	693 (240–1386)	0.315
Sedentary index	1680 (840–2100)	2100 (787.5–3990)	1680 (840–2100)	0.343
Income, median (IQR)	600 (369–750)	503 (366–765.25)	3000 (370–750)	0.615
Arterial hypertension_Yes, *n* (%)	11 (10.3)	1 (12.5)	10 (10.1)	0.593
Diabetes mellitus_Yes, *n* (%)	14 (13.1)	1 (12.5)	13 (13.1)	1.000
Dyslipidemia_Yes, *n* (%)	19 (17.8)	2 (25)	17 (17.2)	0.630
FEV1 *z*-score	−1.41 (1.22)	−2.7 (1.0)	−1.31 (1.18)	0.002
FVC *z*-score	−0.99 (1.16)	−2.03 (0.94)	−0.91 (1.14)	0.012
FEV1/FVC *z*-score	0.88 (0.99)	−1.71 (0.95)	−0.81 (0.96)	0.019
FEV1 *z*-score severity, *n* (%)				0.043
Normal	60 (56.1)	1 (12.5)	59 (59.6)	
Mild	26 (24.3)	3 (37.5)	23 (23.2)	
Moderate	20 (18.7)	4 (50)	16 (16.2)	
Severe	1 (0.9)		1 (1)	
FVC *z*-score severity, *n* (%)				0.012
Normal	78 (72.9)	2 (25)	76 (76.8)	
Mild	17 (15.9)	4 (50	13 (13.1)	
Moderate	11 (10.3)	2 (25)	9 (9.1)	
Severe	1 (0.9)		1 (1%)	
FEV1/FVC *z*-score severity, *n* (%)				0.059
Normal	82 (76.6)	4 (50)	78 (78.8)	
Mild	18 (16.8)	2 (25)	16 (16.2)	
Moderate	8 (6.5)	2 (25)	5 (5.1)	

Abbreviations: FEV1, forced expiratory volume; FEV1/FVC, ratio forced expiratory volume/forced vital capacity; FVC, forced vital capacity; METS, metabolic equivalents of task.

**Figure 1.**
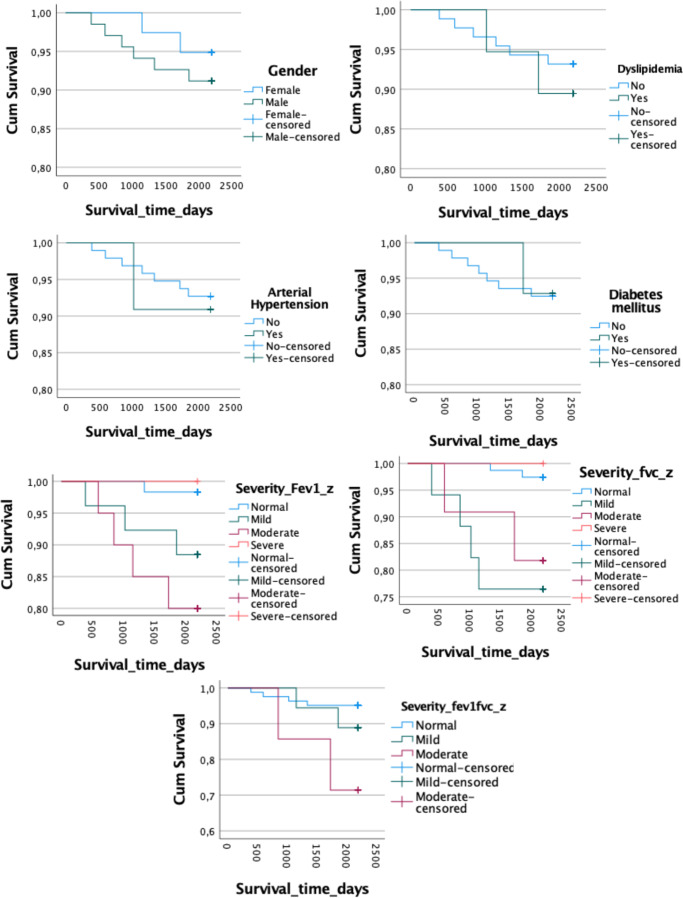
Kaplan–Meier curves. FEV1, forced expiratory volume; FEV1/FVC, ratio forced expiratory volume/forced vital capacity; FVC, forced vital capacity.

The Kaplan–Meier curves revealed no significant differences in survival time between the deceased group and survivors for gender (log-rank test, *χ* ^2^ = 0.510, *p* = 0.475) or the presence of hypertension (log-rank test, *χ* ^2^ = 0.049, *p* = 0.825), diabetes (log-rank test, *χ* ^2^ = 0.001, *p* = 0.940), dyslipidemia (log-rank test, *χ* ^2^ = 0.288, *p* = 0.521), or FEV1/FVC *z*-score severity (log-rank test, *χ* ^2^ = 5.832, *p* = 0.054). However, survival time differed significantly based on FEV1 *z*-score severity categories (log-rank test, *χ* ^2^ = 8.384, *p* = 0.039), and FVC severity (log-rank test, *χ* ^2^ = 11.972, *p* = 0.007; Table 2 and Figure 1).

**Table 2.** Kaplan–Meier curves

	*N* total	Events	Censored (%)	Mean survival time (days)	95% CI for mean	Log-rank (Mantel–Cox) chi-square	*p*-value
Gender							
Female	39	3	37 (94.9)	2151.400	2095.45–2207.37		
Male	69	6	99 (92.5)	2085.529	1998.68–2172.38		
Overall	107	8	99 (92.5)	2109.542	2050.40–2168.69	0.510	0.475
Arterial hypertension							
No	96	7	89 (92.7)	2112.490	2050.64–2174.34		
Yes	11	1	10 (90.9)	2083.818	1885.39–2282.25		
Overall	107	8	99 (92.5)	2109.542	2050.40–2168.69	0.049	0.825
Diabetes mellitus							
No	93	7	86 (92.5)	2102.430	2035.158–2169.702		
Yes	14	1	13 (92.9)	2156.786	2094.054–2219.518		
Overall	107	8	99 (92.5)	2109.542	2050.397–2168.687	0.006	0.940
Dyslipidemia							
No	88	6	82 (93.2)	2110.727	2043.97–2177.49		
Yes	19	2	17 (89.5)	2104.053	1980.26–2227.85		
Overall	107	8	99 (92.5)	2109.542	2050.40–2168.69	0.288	0.591
FEV1 *z*-score severity				*			
Normal	60	1	59 (98.3)				
Mild	26	3	23 (88.5)				
Moderate	20	4	16 (80)				
Severe	1	0	1 (100)				
Overall	107	8	99 (92.5)			8.384	0.039
FVC *z*-score severity				*			
Normal	78	2	76 (97.4)				
Mild	17	4	(13 (76.5)				
Moderate	11	2	9 (81.9)				
Severe	1	0	1 (100)				
Overall	107	8	99 (92.5)			11.972	0.007
FEV1/FVC *z*-score severity							
Normal	82	4	78 (95.1)	2123.87	2058.23–2189.50		
Mild	18	2	16 (88.9)	2113.55	1999.91–2227.20		
Moderate	7	2	5 (71.4)	1931.43	178.27–1582.02		
Overall	107	8	99 (92.5)	2109.54	2050.40–2168.69	5.832	0.054

Abbreviations: FEV1, forced expiratory volume; FEV1/FVC, ratio forced expiratory volume/ forced vital capacity; FVC, forced vital capacity.

The significance is in the “overall” row at the same of the others variables.

The univariate Cox regression analyses revealed that lower FEV1 *z*-score (HR = 0.407, 95% confidence interval [CI]: 0.229–0.721, *p* = 0.002), FVC *z*-score (HR = 0.452, 95% CI: 0.248–0.823, *p* = 0.009), and abdominal circumference (HR = 1.065, 95% CI: 1.02–1.112, *p* = 0.005) were significantly associated with an increased mortality risk in individuals with SMI, while age showed a nonsignificant trend toward an association with mortality (HR = 1.105, 95% CI: 0.982–1.233, *p* = 0.089). Other variables included in the univariate Cox regression (number of cigarettes, pack/year, activity index [METS], sedentary index, and income) were not significantly associated with mortality in our sample (Table 3).

**Table 3.** Univariate Cox regression

	Coef (B)	SE	*p*-value	HR (EXP (B))	95% CI para HR
Age	0.100	0.060	0.097	1.105	0.982–1.244
Cigarettes per day	−0.008	0.035	0.816	0.992	0.925–1.063
Pack/year	0.014	0.016	0.399	1.014	0.982–1.047
Activity index (METS)	0.000	0.000	0.671	1.000	0.999–1.001
Sedentary index	0.000	0.000	0.155	1.000	1.000–1.001
Abdominal circumference	0.063	0.022	0.005	1.065	1.020–1.112
Income	0.000	0.001	0.697	1.000	0.998–1.001
FEV1 *z*-score	−0.900	0.202	0.002	0.407	0.229–0.721
FVC *z*-score	−0.794	0.306	0.009	0.452	0.248–0.823

Abbreviations: FEV1, forced expiratory volume; FVC, forced vital capacity; METS, metabolic equivalents of task.

We constructed two Cox regression models to avoid multicollinearity based on the significant association observed in the univariate analyses. Each model included age, abdominal circumference, and one of the following lung function parameters: FEV1 *z*-score or FVC *z*-score (Table 4). Both models demonstrated a statistically significant overall fit, as indicated by the omnibus test of model coefficients (FEV1 model: *χ* ^2^ = 14.95, df = 3, *p* = 0.002; FVC model: *χ* ^2^ = 12.64, df = 3, *p* = 0.005). However, only the model with FEV1 demonstrated a statistically significant association between lower FEV1 (Coef (B) -0.748) and increased mortality risk (HR = 0.473, 95% CI: 0.220–0.979, *p* = 0.044).

**Table 4.** Multivariate models: Cox regression

	Coef (B)	SE	*p*-value	HR (EXP (B))	95% CI para HR
Model with FEV1 z-score					
Age	0.131	0.072	0.068	1.140	0.991–1.312
Abdominal circumference	0.029	0.024	0.216	1.030	0.938–1.079
FEV1 *z*-score	−0.748	0.370	0.044	0.473	0.229–0.979
Model with FVC *z*-score					
Age	0.119	0.072	0.097	1.126	0.979–1.296
Abdominal circumference	0.041	0.025	0.102	1.042	0.992–1.095
FVC *z*-score	−0.519	0.350	0.138	0.595	0.229–1.182

Abbreviations: FEV1, forced expiratory volume; FVC, forced vital capacity.

## Discussion

To our knowledge, this is the first study to evaluate the predictive capacity of lung function on all-cause mortality in individuals with SMI, a population at high risk for premature mortality. In the prospective analysis of a clinical sample of middle-aged smokers with SCHIZ and BD, the lung function parameter FEV1 predicted all-cause mortality. Specifically, a lower baseline *z*-score FEV1 showed a significantly increased risk of death, adjusting with well-known mortality risk predictors, such as age, sex, abdominal circumference, hypertension, dyslipidemia, and diabetes.

The present results align with numerous previous studies that have demonstrated an association between reduced lung capacity and mortality risk in the general population [27–30]. Although FVC has also been described as a robust predictor of mortality in the general population [31], FEV1 is considered a better predictor [32, 33], even with a higher predictive power than blood pressure or body mass index (BMI) [34]. It is predictive even with mild-to-moderate volume reductions. It retains its effectiveness in nonsmoking populations, young individuals (under 50 years old), and healthy participants [16], independent of other mortality risk factors, including cardiovascular ones [35].

People with SMI are overexposed to a lifetime context of physical health risks, starting from intrauterine life throughout adulthood, which may also impact lung development and respiratory health. SMI is associated with an increased likelihood of prenatal tobacco exposure, low birth weight, preterm births, and early-life adversities [36] – determinant factors in reaching peak FEV1 in early adulthood, a central component in the current paradigm of respiratory health [17, 37]. Tobacco is the leading preventable mortality factor and the critical element in the reduction of FEV1 in people with BD and SCHIZ. These individuals have smoking rates of between 45 and 65%, respectively, younger onset, more intense smoking – depth and frequency of puffing – and a higher level of dependence [18]. Other risk factors contribute to completing the complex network of environmental factors leading to a reduction in FEV1 and poor lung function among people with SMI: alcohol consumption, sedentary lifestyle, unhealthy dietary habits, metabolic syndrome, and poorer living conditions [38].

Despite the consistent relationship between respiratory diseases and SMI, respiratory function studies in that population are still scarce. However, in the last decade, three clinical samples of SCHIZ – one included BD – were studied by spirometry [11, 13, 39] and one was extracted from a population database with SCHIZ and other nonaffective psychoses [40]. Collectively, these studies confirm a higher frequency of altered respiratory patterns, with reductions in FEV1 and FVC, and their possible link with several risk factors, mainly to smoking, abdominal circumference, and metabolic syndrome. The longitudinal follow-up of one of the samples alerts suggests the possibility of an accelerated FEV1 decline in the first 3 years after detection, well above that described in the general population [41]. Moreover, physical activity acted as a possible protective factor [41].

In addition to the reduction in lung volumes, recent meta-research confirms that the risk of lung diseases, mainly chronic obstructive pulmonary disease (COPD), is increased in the SMI population and especially in young people [42]. Follow-up studies in high-income countries confirm high mortality rates due to COPD, with the risk being up to 11 times higher in individuals with SCHIZ and those under 50 years of age compared to the general population [7]. Recently, in a community sample of individuals with SCHIZ and BD, with a mean age of 49 years and no previous respiratory symptoms, one in four patients was diagnosed with COPD, 80% of whom were at moderate or severe stage [18].

The mechanisms linking low FEV1 to mortality are complex and not yet fully understood. Based on previous evidence from the general population, reduced FEV1 may appear within an obstructive (FEV1/FCV < LLN ratio) and pre-obstructive (preserved ratio) pattern, and thus is mainly linked to respiratory, cardiovascular, and oncologic pulmonary mortality [43]. In those patterns with preserved ratio and FVC < LLN, reduced FEV1 would be associated with mortality through metabolic disease and mainly cardiovascular and nonpulmonary oncologic risk [43]. In samples of smokers, such as the one we present, the relationship with mortality may also occur directly through the specific risks associated with smoking, and in all cases, through the link with the multiple risk factors previously described (unhealthy habits, physical inactivity, and socioeconomic determinants), which affect both overall physical health and respiratory health.

To date, results on the motivational value of providing spirometry test information for smoking cessation have been scarce and inconclusive in the general population. However, promising results have recently been described in a pilot study in which lung age was reported to smokers, who subsequently demonstrated high cessation rates at 12 weeks [44]. Specifically in BD and SCHIZ, a controlled clinical trial that analyzed the value of spirometry-derived lung age information as a motivational tool demonstrated a significant reduction in the number of cigarettes/day and the biological measure of expired CO levels, as well as an increased likelihood of smoking cessation at 12 weeks [18].

The evidence linking lung function and mortality risk is robust in the general population. The factors threatening respiratory health from lung development and through adulthood are common to those observed for other chronic diseases [16], and the reduction in FEV1 may reflect a common, multiorgan inflammatory context [45]. In any case, until there is a better understanding of these mechanisms, it seems that lung function – mainly through the reduction of FEV1 – could serve as “the canary in the coal mine,” alerting us to a broader risk to physical health. A simple, inexpensive, and therefore implementable test in the community care setting would permit identifying individuals with SMI at greater physical health risk and developing more intensive, individualized prevention actions of greater intensity. Such efforts should target mainly smoking cessation, but also the other highlighted risk factors.

Despite the strength of being the longitudinal design and of being the first study of its kind in an SMI sample looking into the observed independent predictive power of FEV1, some limitations should be considered when interpreting the present results. First, the relatively small sample size and lack of statistical power may have underestimated the true predictive power of FVC [46]. It also limited the ability to discriminate mortality risk levels based on spirometry patterns and the presence or absence of respiratory symptoms, as well as its predictive value for the specific cause of death. Second, the variables analyzed were those measured at baseline, which did not allow us to assess the potential influence of a wider range of lifestyle factors and lung volume evolution on the mortality outcome [47]. Moreover, treatment was naturalistic, and its influence cannot be estimated [48].

In summary, in a population at high risk of premature mortality, such as individuals with SMI, it is a priority to develop initiatives for a timely diagnosis and prevention. Longitudinal follow-up of larger samples will help confirm the results of lung damage described so far in the literature and those presented in this study regarding the predictive value of lung function on mortality. Deepening into the age ranges at greatest risk, severity levels depend on the degree of FEV1 reduction and the predictive value on cause-specific mortality. If replicated, spirometry could be an opportunity to diagnose COPD on time and identify individuals at a higher overall risk of mortality, enabling targeted, intensive, and individualized interventions.

## Data Availability

Data are available from the corresponding author upon request.
